# Mild heat induces a distinct “eustress” response in Chinese Hamster Ovary cells but does not induce heat shock protein synthesis

**DOI:** 10.1038/s41598-017-15821-8

**Published:** 2017-11-15

**Authors:** Begüm Peksel, Imre Gombos, Mária Péter, László Vigh, Ádám Tiszlavicz, Mario Brameshuber, Gábor Balogh, Gerhard J. Schütz, Ibolya Horváth, László Vigh, Zsolt Török

**Affiliations:** 1 Institute of Biochemistry, Biological Research Centre of the Hungarian Academy of Sciences, Szeged, H-6726 Hungary; 2 Institute of Applied Physics – Biophysics, TU Wien, 1040 Vienna, Austria

## Abstract

The current research on cellular heat stress management focuses on the roles of heat shock proteins (HSPs) and the proteostasis network under severe stress conditions. The mild, fever-type stress and the maintenance of membrane homeostasis are less well understood. Herein, we characterized the acute effect of mild, fever-range heat shock on membrane organization, and HSP synthesis and localization in two mammalian cell lines, to delineate the role of membranes in the sensing and adaptation to heat. A multidisciplinary approach combining ultrasensitive fluorescence microscopy and lipidomics revealed the molecular details of novel cellular “eustress”, when cells adapt to mild heat by maintaining membrane homeostasis, activating lipid remodeling, and redistributing chaperone proteins. Notably, this leads to acquired thermotolerance in the complete absence of the induction of HSPs. At higher temperatures, additional defense mechanisms are activated, including elevated expression of molecular chaperones, contributing to an extended stress memory and acquired thermotolerance.

## Introduction

Stress response is a vitally important biological process that protects organisms against various environmental and pathophysiological conditions. Interestingly, the core of this ancient protective process is very similar in all living species^1^. Already in 1976, Hans Selye distinguished different levels of stress. He termed mild stress “eustress” and commented that such stress was often beneficial, leading to enhanced resilience and performance^2^. As the stress level increases, however, it will cause “distress”, which can be harmful or even lethal. The cellular stress response comprises a very complex arsenal of defense mechanisms, and depends not only on the stressor itself but also on the level and extent of the stimuli, and the subject of the stress. Therefore, defining and dissecting different levels of this crucial process is critically important.

Based on the intensity of stress, the heat stress (HS) is currently classified as mild or severe. This classification is not precise since the sensitivity to heat varies depending on such multiple factors as cell type, developmental stage, cell cycle phase, the dose (duration and temperature) of stress, and the actual parameters measured. The mild, fever-ranged (38–41.5 °C) non-proteotoxic HS is one of the most frequent functional perturbations in most life forms, especially in homeothermic animals; thereby, understanding the HS sensing mechanisms and the different cellular response approaches is especially important.

Currently, HSP induction and cellular redistribution of pre-existing HSPs are considered to be an essential part of the stress defense mechanisms at all stress levels^3–5^. The orchestration of heat shock protein response is a rapid, highly regulated cellular process, which involves a coordinated control of multiple signal transduction pathways, including multiple kinase cascades (JNK/SAPK, p38/HOG1, and ERKs). The central role of heat shock factor 1 (HSF1) in cellular homeostasis is well known: its activation is required for inducible HSP expression and acquired thermotolerance (ATT) in mouse embryonic fibroblasts^6^.

Considerable evidence has accumulated in favor of the “membrane sensor (thermometer) hypothesis”, which predicts that plasma membrane alterations affect the extent of HSP activation^7,8^. This is especially pertinent to mild HS, a condition present during fever in vertebrates^8–10^. It is conceivable that HS sensor(s) evolved in parallel to the fever response in vertebrates; such HS sensor(s) would detect the elevation of temperature via membrane thermometers, in the absence of any recognizable damage to the main cellular functions, forming the evolutionary basis for the membrane sensor theory. Stressful conditions activate numerous membrane-associated sensors residing at the top of interconnected signaling pathways that are shaped by the chemical and physical state of the membrane^8,11^.

Membrane glycerophospholipids, sphingolipids (SLs), and cholesterol are involved in the generation of stress-induced secondary messengers^7^. Precise identification and quantitation of the cellular lipids is therefore prerequisite for mapping the interactions and dynamics of membrane lipids during stress response. The recent advances in mass spectrometric (MS) techniques have facilitated the development of lipidomics, permitting reliable analysis of multiple lipid species within short analysis time. Lipidomics of the yeast stress response uncovered the essential function of SLs in heat protection, coupled to their signaling role in HSP production^12^. Our recent investigation of *Schizosaccharomyces pombe* revealed the existence of a metabolic crosstalk between membrane and storage lipids in response to HS; this crosstalk facilitates the homeostatic maintenance of the physical/chemical state of the membrane, preventing negative effect of HS on cell growth and viability^13^. The key consequences of the membrane lipid-controlled sensing and signaling of HS include membrane lipid rearrangement, generation of signaling lipids via activation of various lipases, and *de novo* synthesis of mediator-type lipids. These have been recently reviewed in various yeast, plant, and mammalian species^11–16^.

In recent years, our understanding of the plasma membrane (PM) has changed considerably, as the knowledge of membrane microdomains has expanded; nevertheless, a universal model of lateral organization of the highly dynamic PM is still missing^17^. One of the most popular membrane model describes “lipid rafts” as high molecular order PM platforms, enriched in cholesterol and SLs, where proteins involved in signaling and the effector molecules may selectively interact^18^. Since lipid microdomains have been widely shown to play important roles in the compartmentalization, modulation, and integration of cell signaling^19^, these nanoplatforms may additionally play an influential role in stress sensing and signaling. Since the raft structure depends on the thermally affected behavior of the lipid phase, even mild changes in temperature could result in a fundamentally altered solubility and, consequently, redistribution, of signaling proteins in the rafts.

In the current study, we used chinese hamster ovary (CHO) and mouse embryonic fibroblast (MEF) cells to dissect the HS into well-defined stress levels, characterized by specific HSR. Our ultrasensitive fluorescence microscopy and lipidomics experiments revealed the molecular details of a novel cellular “eustress”, when cells adapt to mild heat by maintaining membrane homeostasis, activating lipid remodeling, and redistributing chaperone proteins leading to acquired thermotolerance in the absence of the induction of HSPs.

## Results

### The regulation of HSPs depends on the heat dose

**Figure 1:**
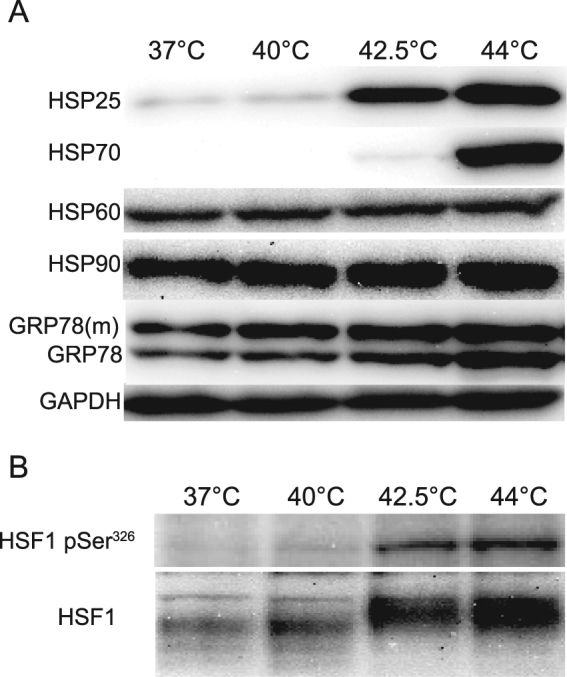
The effect of temperature on HSP induction and HSF1 activation. (**A**) A GPI-mGFP–expressing CHO cell line was incubated for 20 min at the specified temperatures; the samples were prepared for western blotting after 6 h of recovery at 37 °C. (**B**) HSF1 phosphorylation was analyzed in samples after 20 min of heat treatment without recovery. See Supplementary Figure S5 and S6 for the full images of Fig. 1A,B, respectively.

HSP induction is highly dependent on the level and duration (dose) of the heat treatment. To assess the minimal dose required to induce HSPs in CHO cells, we used immunoblotting to systematically analyze HSP levels following heat treatment at different temperatures (Fig. 1, Supplementary Fig. S1). To be able to compare the results with image-based fluorescence correlation spectroscopy (ImFCS) studies (Fig. 5), we employed a CHO cell line expressing glycosylphosphatidyl-inositol-anchored monomeric green fluorescent protein (GPI-mGFP)^20^. Out of the five major HSPs examined, only HSP25, HSP70, and GRP78 were induced by moderate (42.5 °C) and severe (44 °C) heat; no HSP induction was observed when the cells were treated with mild heat (40 °C) for 20 min (Fig. 1). The different threshold temperatures for a substantial increase in HSP25 (42.5 °C) and HSP70 (44 °C) levels suggested a differential regulation of these two proteins. HSP25 induction was the most heat-sensitive of the two. Next, we analyzed the duration of HS required before HSP induction becomes detectable (Supplementary Fig. S1). While 40 °C did not induce HSPs for up to 40 min, 10 min at 42.5 °C resulted in a pronounced increase in HSP25 levels, followed by their further, gradual increase. HSP70 induction showed a biphasic profile, with a weak induction in the first 20 min of HS, followed by a marked response after 40 min. Similarly, a notable induction of HSP90 and GRP78 was observed after 40 min. Consequently, to maintain the minimal level of stress sufficient to trigger HSP induction (at 42.5 °C), the 20 min heat treatment was selected for all further experiments.

HSF1, as the major orchestrator of HSP induction, is phosphorylated before it interacts with heat shock elements to trigger the expression of HSPs^21^. Therefore, we investigated the overall phosphorylation of HSF1 and also its specific phosphorylation at Ser^326^; the latter was shown to be strongly correlated with stress-induced transactivation competence^22^. In these experiments, samples were collected after a 20 min heat treatment. The molecular mass of HSF1 has increased at 42.5 °C and 44 °C, but not at 40 °C, which was similar to the HSP induction data and could be the result of phosphorylation of HSF1 as reflected by Ser^326^ phosphorylation (Fig. 1B).

### Redistribution of HSPs upon heat treatment

**Figure 2:**
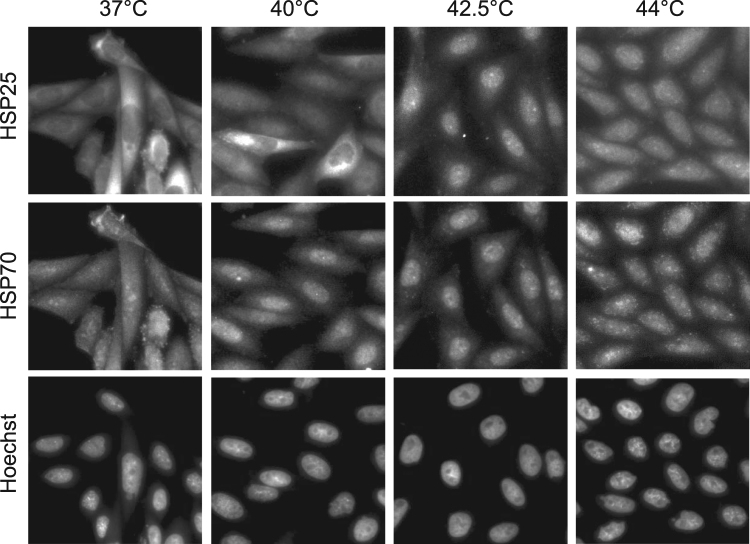
The effect of heat treatment on the intracellular localization of HSPs in CHO cells. Representative images (n = 40) of CHO cells after a 20 min heat treatment at the indicated temperatures are shown.

As part of the HSR, pre-existing HSPs are re-distributed within the intracellular compartments to carry out protective and preventive activities^4^. Hence, following the HSP re-localization is important for understanding the heat dose-dependent response, while allowing a visual inspection/assessment of the early HSR quickly post/into the heat treatment. We performed immunostaining experiments to investigate the localization of the most heat-responsive HSPs, HSP25 and HSP70, after 20 min of treatment at 37 °C, 40 °C, 42.5 °C, and 44 °C (Fig. 2). The two HSPs translocated to the nucleus at 40 °C and 42.5 °C in a temperature-dependent manner. Single-cell analysis revealed a large cell-to-cell variation in the redistribution of the two HSPs at 40 °C and 42.5 °C, with a much higher heterogeneity in the case of HSP25. HSP70 translocation was more sensitive to a mild temperature change. This suggested the existence of another level of regulation of the cellular stress protein response, which controls the localization of a preexisting HSP pool; it would constitute an immediate response, probably protecting the most vulnerable cellular functions in the nucleus. These experiments also revealed that the HSP distribution pattern at 44 °C is different from that observed at lower temperatures; namely, the two HSPs showed a perinuclear accumulation, with enrichment in perinuclear spots. Heat-dependent HSP translocation displayed similar pattern in mouse embryonic fibroblast (MEF) cells (Supplementary Fig. S3).

### Mild “eustress” induces ATT in the absence of HSP synthesis

**Figure 3:**
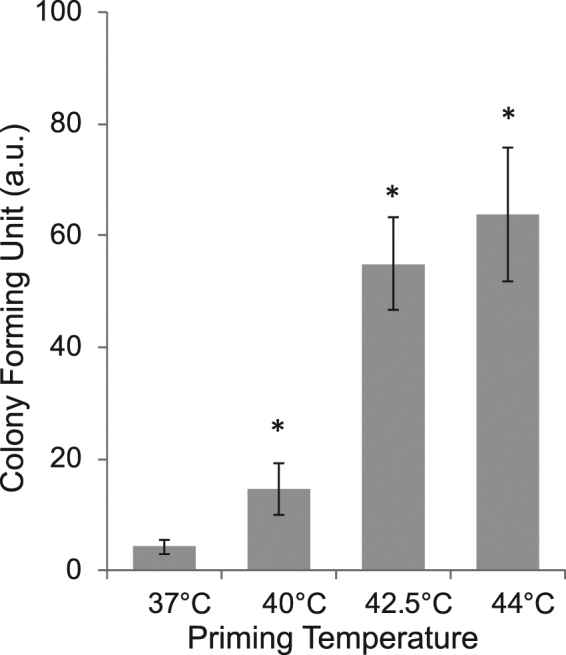
ATT of CHO cells primed with different heat doses. A quantitative analysis of the results of colony formation assay is shown. CHO cells were primed for 20 min at the specified temperatures; after a 6 h recovery at 37 °C, the cells were exposed to 46 °C for 20 min. The mean values were normalized to controls which were kept at 37 °C throughout the duration of the experiments (n = 3; error bars represent SEM; *p < 0.05 indicates significance vs. time 0, Student’s *t*-test).

In prokaryotes, yeast, and *Drosophila*, the accumulation of heat shock proteins correlates with increased thermotolerance; however, in higher eukaryotes, the existence of such association is controversial^23,24^. Therefore, we next addressed the question whether cells that experience mild stress without HSP induction are nonetheless able to acquire thermotolerance. CHO cells were exposed to priming heat for 20 min and then, after a 6 h recovery at 37 °C, to a lethal heat treatment, at 46 °C for 20 min; cell survival was next assessed by the colony formation assay (Fig. 3). Pre-treatment at 40 °C resulted in a significant increase (p < 0.05) in cell survival, in spite of the absence of HSP induction (Fig. 1). Priming the cells at 42.5 °C and 44 °C led to the development of a significantly higher thermotolerance than priming at 40 °C (Fig. 3; p < 0.05), in parallel with a pronounced induction of HSPs (Fig. 1); however, it was difficult to link ATT with HSP70 levels. Hence, heat-induced ATT is highly dependent on the dose of the initial heat treatment but only weakly linked to HSP induction.

**Figure 4:**
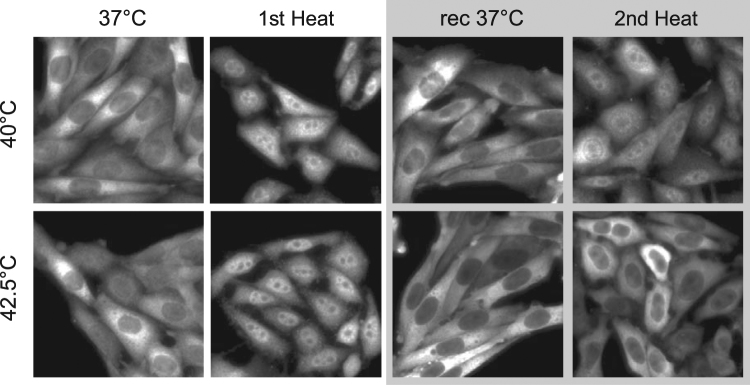
HSP25 distribution after two consecutive heat treatments of CHO cells. Representative images (n = 38) of HSP25 distribution in CHO cells at 37 °C, after a 20 min heat treatment at specified temperatures (1st heat); followed by 6 h recovery at 37 °C (rec 37 °C); and after a second, 20 min, heat treatment at specified temperatures (2nd heat).

Since translocation of HSP25 and HSP70 to the nucleus was transient at all investigated temperatures, and a return to normal distribution was observed after a 60 min recovery at 37 °C (data not shown), it could not have contributed to increased ATT. To investigate whether ATT manifests at the occurrence of HSP redistribution, we next asked how the redistribution of HSP25 is affected by a 20 min priming at higher temperature. The cells were exposed to two identical and consecutive heat treatments, separated by a 6 h recovery period. After the second heating cycle, HSP25 re-localized to the nucleus at 40 °C, however no nuclear enrichment was observed in CHO (Fig. 4) or MEF (Supplementary Fig. S3) cells at 42.5 °C.

### Cells promptly respond to heat by remodeling their PM

The lateral diffusion and confinement properties of fluorescent membrane probes were next followed by ImFCS to monitor the heat-induced changes in the structure and dynamics of PM. This method enables real-time spatial multiplexing of FCS measurements, with relatively high temporal resolution (ms); it also allows post-acquisition binning of pixels for the calculation of autocorrelation functions in multiple observation areas from single measurements^25^. Diffusion coefficient (D) and confinement time (τ_0_, the y-intercept in the graph of the FCS diffusion laws) describe the lateral diffusion of the probe and the organization of the membrane, respectively^26^. For a heterogeneous membrane, τ_0_ reflects the convolution of three diffusion modes: hop diffusion (τ_0_ < 0), free (τ_0_ = 0) and confined (τ_0_ > 0) diffusion^27^.

**Figure 5:**
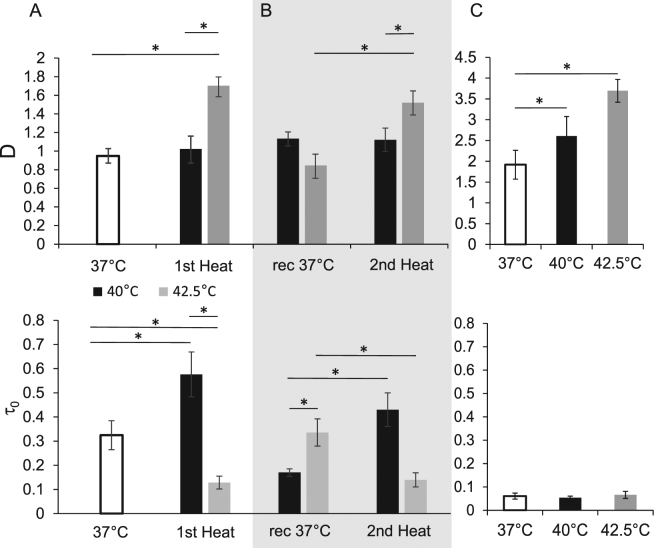
The alteration of membrane diffusion of GPI-mGFP in live cells at different heat exposures. The diffusion coefficient (D) and confinement time (*τ*_0_) of GPI-mGFP on the surface of CHO cells were measured by ImFCS (**A**) during a 15 min primary treatment (1st heat) at 37 °C, 40 °C, and 42.5 °C; (**B**) after a 6 h recovery at 37 °C (37 °C), and upon a 15 min second heating cycle (2nd heat) at the same temperature as in (A). (**C**) ImFCS analysis of PM sheets isolated from CHO cells, at 37 °C, 40 °C, or 42.5 °C. The values represent averages, and the error bars represent the standard error of mean (SEM) (n = 8, p < 0.05).

The CHO cell line used in the current study constitutively expresses the liquid-order membrane domain reporter GPI-mGFP, enabling the determination of membrane diffusion properties. In live cells, the D of GPI-mGFP in the PM significantly increased (p < 0.01) at 42.5 °C; unexpectedly, it remained unchanged upon heat treatment at 40 °C (Fig. 5A). At 44 °C, the cells started to lift from the cover slide, rendering the total internal reflection fluorescence (TIRF) microscopy impossible. The positive τ_0_ indicated a confined diffusion of GPI-mGFP at all measurement temperatures.

Several studies on GPI-anchored proteins, such as ones utilizing homo-fluorescence resonance energy transfer-based fluorescence anisotropy measurements^28,29^, near-field scanning microscopy^30^, photo-activation localization microscopy^31^, and single molecule microscopy^20^, revealed that ca.20–40% of GPI-anchored proteins are present as nanoclusters on membranes; the rest are monomeric, and continuously in exchange with the relatively immobile nanoclusters^28,32^. The observed positive confinement time might have been associated with these (or larger association of) nanoclusters^17,33,34^. HS affected τ_0_ in an opposite manner, depending on the level of stress. While the confinement remarkably increased at 40 °C, it decreased at 42.5 °C, compared to 37 °C (Fig. 5A). Since the regulation and the nucleation of GPI-mGFP clusters are active cellular processes [e.g., involving transient attachment between PM and cortical actin], analogous experiments were performed with isolated PM sheets. Preparation of the membrane sheets by sonication ensured the absence of cortical actin (data not shown), hence, the effects of cortical actin and any active cellular process were eliminated. Compared to live cells, in membrane sheets, the D was two times higher, and the τ_0_, was six times lower at 37 °C (Fig. 5C). The consistent temperature-dependent increase of the D parameter and the unaltered τ_0_ in PM sheets clearly suggests that the observed changes of diffusion at 40 °C in live cells are a consequence of active processes, such as altered enzymatic reactions and/or vesicular lipid exchange.

To assess the manifestation of heat-induced ATT at the membrane structure level, the effect of a second identical heat treatment after a 6 h recovery at 37 °C was examined (as in the HSP25 redistribution experiment, see Fig. 4). The heat-induced changes in D and *τ*_0_ parameters were similar to those after the first heating cycle (Fig. 5B). On the other hand, after recovery from 40 °C, the GPI-mGFP diffusion was significantly less confined (p < 0.05) than before the first heating cycle (i.e., smaller *τ*_0_).

To validate the results obtained with the lipid-anchored protein probe, the FCS experiments were repeated using a lipid probe Bodipy FL-SM; similarly to GPI-mGFP, it is thought to have a preference for liquid-order domains^35^. While this probe exhibited a significantly higher confinement (i.e., larger *τ*_0_), the heat treatments led to essentially the same relative diffusion and confinement time changes as in the case of GPI-mGFP (Fig. 5, Supplementary Fig. S4).

### Lipidomics analysis reveals distinct stress lipidomes

To test the effect of heat on the lipid composition of GPI-mGFP–expressing CHO cells, we used comprehensive electrospray ionization MS to analyze total membrane lipid extracts. Lipids were isolated after treating the cells at different temperatures for 20 min. The high-sensitivity, high-resolution shotgun lipidomics approach allowed the identification and quantitation of ca. 160 molecular species, encompassing 15 lipid classes.

**Figure 6:**
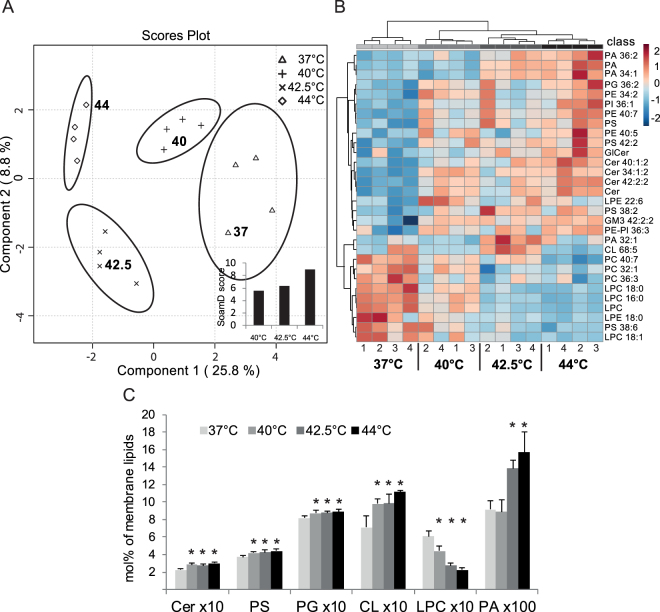
Characterization of lipidomes of GPI-mGFP–expressing CHO cells exposed to different levels of HS. (**A**) sPLS-DA and SoamD score (inset) of lipid molecular species. (**B**) Heatmap representation of hierarchical clustering of lipid profiles at 37 °C, 40 °C, 42.5 °C, and 44 °C. The top 30 most significant species were selected using ANOVA, Euclidean distance, and clustering algorithm Ward. The heat color-code represents normalized values (z-scores), ranging between [−2] (darkest shade of blue) and [+2] (darkest shade of red). Data from four independent experiments with control and HS-treated cells are shown. (**C**) Key features of HS-associated lipid remodeling of CHO lipidomes: ceramide (Cer), phosphatidylserine (PS), phosphatidylglycerol (PG), cardiolipin (CL), lysophosphatidylcholine (LPC), and phosphatidic acid (PA). The cells were either unstressed (37 °C) or stressed at 40 °C, 42.5 °C, or 44 °C, for 20 min. The values are expressed as mol% of membrane lipids (mean +SD), n = 4; *p < 0.05, (vs. 37 °C).

To obtain an overview of lipidome changes, all MS data (expressed as mol% of membrane lipids) were subjected to statistical analysis. After the sparse partial least squares discriminant analysis (sPLS-DA), the different temperature groups (37 °C, 40 °C, 42.5 °C, and 44 °C samples) were very well separated (Fig. 6A). The sum of absolute mol% difference relative to control (SoamD score)^36^ was then employed to rank the stress lipidomes of CHO cells. This approach revealed gradually increasing changes in the global lipid composition with an increasing stress temperature (Fig. 6A inset). The data were further analyzed by comparing the molecular species patterns in individual sample types; almost 180 statistically significant differences were identified (including all pairwise comparisons with the control, p < 0.05; Supplementary table S1). The heatmap representation of hierarchical cluster analysis reflected the distinctly different lipid patterns in all experimental conditions (Fig. 6B). Under mild (40 °C) and moderate (42.5 °C) stress conditions, ca. 30% of the lipidome changed significantly. The greatest extent of significant alterations (ca. 50% of the lipidome) was observed under severe stress conditions (44 °C), when the gradually accumulating changes in abundance (either elevation or reduction) of several lipid species exceeded the significance threshold (p < 0.05, Supplementary table S1). It should be noted that these significant changes were accompanied by moderate fold changes (between 0.2- and 4.6-fold changes).

To identify the alterations of explanatory relevance in stress response, the data were analyzed in more detail. At lipid class level, the increase of ceramide (Cer) abundance was significant (p < 0.05) under all stress conditions (Fig. 6C). It is remarkable that the major long chain fatty acid (FA)-containing species (i.e., 42:2:2 species with 24:1 FA) significantly accumulated not only in Cer but also in upstream glycosphingolipid classes, glucosylceramide (GlCer) and GM3 ganglioside (Supplementary table S1). Phosphatidylserine (PS), as well as phosphatidylglycerol (PG), and cardiolipin (CL), also seemed to be common responders with significant increase in abundance elicited by heat insults; on the other hand, the abundance of lysophosphatidylcholine (LPC) was significantly reduced, with the extent of reduction gradually increasing with stress dosage (Fig. 6C). Pairwise comparisons of lipidomics fingerprints at different stress levels revealed that the major difference between mild and moderate stress was manifested in the alterations of PA abundance; it remained unchanged at 40 °C, while showing a marked (50%) enrichment at 42.5 °C and 44 °C (Fig. 6C).

## Discussion

Based on their HSP response, we herein identified three distinct heat doses for CHO and MEF cells, namely: mild (40 °C; eliciting a novel eustress without HSP induction), moderate (42.5 °C), and severe (44 °C) (Fig. 1, Supplementary Fig. S1). The induction of HSPs is a well-characterized stress response, but it happens hours after the perception of stress^37^. Our study has validated rapid stress dose-dependent redistribution of HSPs as a reliable measure of the stress insult, much closer in time to its onset. Differences in the induction and cellular redistribution of HSPs suggest the operation of strikingly different sensing and signaling mechanisms during these different heat doses. While mild heat does not affect HSF1 phosphorylation and HSPs induction, it initiates the translocation of HSP25 and HSP70 to the nucleus (Fig. 2).The nuclear localization of HSP25 is known to be linked to cytoprotection^38–40^ and was suggested to protect nucleic acids from DNA fragmentation induced by a severe heat shock (43 °C, 3 h), thus preventing apoptotic cell death^41^. Although the entry of HSP27, the human homologue of murine HSP25, into the nucleus was previously shown to strongly correlate with its phosphorylation^38,41^, our results (Supplementary Fig. S2) support the notion that phosphorylation is not required for nuclear translocation of HSP25 during mild and moderate HS, in agreement with the observation that isolated nuclear HSP27 fraction contains non-phosphorylated HSP27 species^41^. Small HSPs, e.g., HSP25, are real molecular factotums with multiple moonlighting functions during stress. Apart from their classical anti-apoptotic chaperone function, they can also interact with membranes, stabilizing the membrane structure and rescuing heat-denatured membrane-associated proteins (reviewed in Horvath *et al*.^5^). The role of nuclear translocation of HSP70 under mild, eustress conditions cannot be gleaned from previous studies in the lack of defined stress doses. Under moderate and severe HS, however, it stabilizes protein complexes in the cytoplasm and temporarily translocates to the nucleus^4,42–45^. Inside the nucleus, it not only protects thermolabile proteins but also facilitates the storage of unfolded proteins within large insoluble foci. In addition, it ascertains the protection of DNA from double-stranded breaks during stress^46–48^.

The induction of thermotolerance at mild, fever-range temperatures, e.g., 39–40 °C, has received little attention thus far. It is remarkable that the mild heat-induced changes reported in the current study lead to an appreciable ATT in CHO cells. This suggests that the cells remember and are prepared for the next stress even without the contribution of newly synthesized HSPs (Fig. 3, Fig. 4, Supplementary Fig. S3).

We define the mild HS described above as a “cellular eustress”. The ability of cells to become more resistant to a second heat shock has previously been linked to HSP production^6^, however, other reports indicated that HSP induction may not be the sole prerequisite for the development of ATT^49,50^. In the current study, no correlation between the development of ATT at 40 °C and HSP synthesis was observed. Although inhibition of HSP synthesis during priming stress was reported to reduce the mean killing time at 45 °C, it does not prevent the development of a certain level of heat tolerance in CHO cells^51^.

It is well established that subtle changes in the physico-chemical properties of surface membranes are the most upstream events of sensing and signaling of mild, fever-type heat stress (for review see Török *et al*.^8^). Experimental data suggest that membrane thermosensing stems from a perturbation of physico-chemical structures of signaling platforms whose specific participants remain unknown^20,52^. To identify specific stress membrane sensors, the sequence of membrane events during the perception of heat should be first dissected.

Heat exposure significantly increases the lateral diffusion of fluorescent membrane probes in the PM of CHO cells at moderate (42.5 °C), but not mild (40 °C), temperatures (Fig. 5; p < 0.05). While the effect of mild heat was successfully balanced by a microdomain rearrangement (i.e., increased domain confinement), moderate heat resulted in both an increased diffusion and reduced confinement of GPI-mGFP (Fig. 5A, Fig. 5B, Supplementary Fig. S4). The increased confinement of GPI-mGFP upon mild HS can be explained by the formation and stabilization of actin-mediated liquid-order lipid nanoclusters^53^, especially in the light of the observed lipidomics changes, discussed below.

Cellular control of the membrane structure during mild HS is an active cell response and/or requires the cortical actin membrane skeleton, since the GPI-GFP diffusion increased, and its confinement decreased, as a function of temperature (as anticipated) in isolated PM sheets lacking cortical actin. The increase of diffusion coefficient value and a significant accompanying decrease of membrane confinement at 42.5 °C (p < 0.05) could result from the uncoupling of cortical actin from the membrane because of deficiencies of cellular responses at higher temperatures. A short severe heat treatment of CHO cells (45.5 °C, 20 min) or a prolonged mild hyperthermia of lymphocytes (39.5–40 °C, 2–12 h) are indeed sufficient to relocalize spectrin (an actin-binding protein that plays an important role in maintaining PM integrity and cytoskeletal structure) from PM into large cytoplasmic aggregates^54,55^, perhaps preventing the formation of actin-mediated membrane nanoclusters^53^.

To allow a comprehensive assessment of the rapid heat-induced changes in the cellular lipid composition of CHO cells, lipidomics datasets were analyzed by using the data-mining sPLS-DA method. This led to an excellent separation of the experiments into four non-overlapping clusters (Fig. 6A). The SoamD scoring algorithm analysis demonstrated that the lipidomes of stressed CHO cells differed from the control; the extent of differences gradually increased with temperature (Fig. 6A inset). Furthermore, a clear separation of all experimental conditions after hierarchical cluster analysis allowed us to conclude that HS treatment gives rise to distinct CHO lipidomic fingerprints, in a dose-dependent manner (Fig. 6B).

The key features of the heat-elicited lipid remodeling, in general, comprised the accumulation of Cer, long chain FA-containing SL species, CL (and PG), PS, and the depletion of LPC (Fig. 6C). The activation of the *de novo* SL synthesis pathway in response to HS is evolutionarily conserved between yeast and mammalian cells^15,56,57^. The elevation in content of long chain FA-containing complex glycoSL species [GlCer(42:2:2) and GM3(42:2:2)] may support the reorganization of PM microdomains upon temperature elevation^53^. Since PS is required for the trans-bilayer coupling and clustering of actin-mediated membrane nanoplatforms^53^, the increased abundance of this lipid class may contribute to the stabilization of the cortical actin-membrane interaction^58–60^. Cholesterol can also stabilize mGFP-GPI nanodomains^17,20,53^, however, we did not detect heat dependent-changes in cholesterol levels under the conditions tested (data not shown). The pronounced decrease in the abundance of LPC (and lysophosphatidylethanolamine) that have strong curvature-inducing and bilayer-destabilizing properties, may also support membrane stabilization^61^. These observations are in agreement with the membrane remodeling detected by ImFCS in the current study (Fig. 5).

CL (and its biosynthetic precursor PG) are marker phospholipids of mitochondria, and their accumulation is coupled to mitochondrial biogenesis. Interestingly, temperature-induced mitochondrial biogenesis was reported in C2C12 myotubes^62^; this would support cellular respiration and might provide energy for the defensive stress response processes.

The increase in phosphatidic acid (PA) abundance is a distinctive feature differentiating the mild and moderate HS (Fig. 6C). PA is an important lipid messenger involved in a wide range of cellular processes, e.g., vesicular trafficking, cytoskeletal organization, secretion, cell proliferation, and cell survival^63^. In mammalian systems, the production of PA by phospholipase D plays a crucial role in the activation of the ERK cascade^64^ and regulation of mTOR signaling^65^, which affects diverse transcription factors, including HSF1^66^. The above literature data are in good agreement with our findings, i.e., that the distinct production of PA at 42.5 °C, but not at 40 °C (Fig. 6C), is paralleled by enhanced activation of several MAPKs (p38 MAPK, SAPK/JNK, and ERK1/2) (data not shown), as well as by HSF1 phosphorylation. Taken together, these results suggest that stress-induced global lipid metabolic reprogramming is co-regulated with adaptive cellular processes. They also highlight the notion that comprehensive lipidomics is a valuable tool complementing membrane biophysics, protein translocation, and stress response signaling data.

How are HS signaling pathways linked to cellular membranes under mild or moderate HS? The described physico-chemical changes in the membranes could lead to altered membrane tension and permeability; lipid and membrane protein rearrangement; changes in transmembrane potential; and the formation of lipid peroxides and lipid adducts^1^. The most likely candidate membrane sensors under these conditions are receptors or receptor networks. The structural changes of members of this putative stress-sensing receptor network could constitute both a rapid signal and an immediate feedback to control the effectiveness of cellular stress response. Nanodomains play a crucial role in triggering the phosphatidylinositol-3 kinase/Akt signaling pathway^67^, which is known to be linked to HSR^68^. Similarly, membrane rafts govern the participation of the small GTPase Rac1 in regulation of HSP expression in B16F10 melanoma cells^69^. All these signaling pathways lead to HSF1 phosphorylation and HSP induction^70^.

The results presented herein clearly show that HSP induction is not critical under short-term mild stress conditions in CHO cells. From the physiological perspective, it seems prudent that the first wave of defense to an acute mild HS should not depend on such time-consuming processes as transcription and translation, but rather, should act primarily by immediately impacting preexisting proteins. The data presented in the current study allow a clear differentiation of a discrete mild eustress, when the cells adapt to fever-type mild heat by maintaining membrane homeostasis, activating lipid remodeling, and redistributing chaperone proteins without inducing HSP synthesis. At higher temperatures, additional defensive mechanisms are activated, including classical HSP expression, which contribute to an extended stress memory and ATT. Further studies are in progress to test the general validity of the above findings in various mammalian systems.

## Materials and Methods

### Cell culture and treatments

CHO (ATCC® CCL-61, Manassas, VA), MEF (ATCC® CCL-61, Manassas, VA), and CHO GPI-mGFP cells^20^ were cultured in 10% FCS supplemented with Ham’s F-12K nutrient mix, and maintained at 37 °C in a humidified 5% CO_2_ atmosphere. The cells were seeded at 250 cells/mm^2^, in 60-mm tissue culture plates (Orange Scientific, Braine-l’Alleud, Belgium) 24 h before the experiments to ensure logarithmic growth during the treatment. For heat shock experiments, the plates were immersed in water baths set to specified temperatures, for the specified duration of time.

### Western blotting

The expression levels of various HSPs were probed with specific antibodies in GPI-mGFP–expressing CHO cells, as follows: HSP25 (ADI-SPA-801, Enzo Life Sciences, Inc., Farmingdale, NY), HSP70 (ADI-SPA-810, Enzo), HSP90 (SMC-107, StressMarq Biosciences, Victoria, BC, Canada), HSP60 (SPC-105, StressMarq), and GRP78 (SPC-180, StressMarq). For the analysis of protein phosphorylation, specific antibodies against phospho-HSP25 (Ser86) (44536, Invitrogen, Carlsbad, CA), phospho-HSP27 (Ser15) (SPA-525, Stressgen, San Diego, CA), phospho-HSF1 (Ser326) (ADI-SPA-902, Enzo), and HSF1 (RT-405, Thermo Scientific, Inc., Waltham, MA) were used. The cells were harvested and lysed in Laemmli sample buffer. Equal protein amounts were measured using Pierce 660 nm protein assay (22660, Thermo Scientific) and loaded on 8% (for HSF1) or 10% SDS gels (for all other determinations) for western blotting experiments^71^ Enhanced chemiluminescence was detected and analyzed using Alpha Ease FC Software v6 (San Jose, CA, USA) after washing and incubating with secondary antibodies IgG peroxidase conjugate (anti-mouse A3682, anti-rabbit A9169, Sigma- Aldrich, Saint Louise, MO). All dilutions were carried out based on the manufacturers' recommendations. Anti-GAPDH (G9545, Sigma-Aldrich) antibody was used to control the protein load and for data normalization.

### Imaging

Multi-color imaging experiments were carried out on wild-type CHO cells. Cells were fixed in 4% (w/v) paraformaldehyde for 10 min immediately after treatment. Cells were permeabilized with 0.5% Triton X-100 for 10 min, and blocked with 1% bovine serum albumin in PBS for 30 min, and incubated with HSP25 (ADI-SPA-801, Enzo) and HSP70 (ADI-SPA-810, Enzo) antibodies. Secondary antibodies from AlexaFluor (A31627, A31570, Thermo Scientific) were selected according to the host species of the primary antibody, and were used at 1:300 dilution, with incubation for 30 min at 37 °C. The cells were washed with PBS three times after each treatment. For the detection of both HSP25 and HSP70, 1:50 antibody dilution was used, with Hoechst 33342 (Thermo Scientific) as a DNA counterstain. Images were taken using Operetta High Content Imaging system using 40× dry objective (PerkinElmer, Inc. Waltham, MA).

### ImFCS

GPI-mGFP–expressing or wild-type CHO cells were seeded into glass bottom dishes (MatTek Corporation, MA) 2 days before the experiments. Wild-type cells were labeled with 50 nM of Bodipy FL C5-sphingomyelin (Molecular Probes, Eugene, OR) for 8 min at 37 °C, and then washed three times with the culture media. The measurements were performed in culture media without phenol red, in a POC-R cell cultivation system (Zeiss, Jena, Germany). This system incorporates a controlled objective heater and CO_2_/air gas chamber. The measurements were initiated after temperature equilibration (ca. 5 min) and completed within 15 min (no appreciable time-dependent changes in the measured parameters were apparent during that time). Before the second heating cycle, the samples were returned to the incubator (37 °C) for a 6 h recovery. Objective-type TIRF illumination was used to achieve the thinnest excited sample volume, with a high numerical aperture objective (alpha Plan-FLUAR 100, Zeiss). The excitation wavelength (488 nm) from a Spectra-Physics Stabile 2018 laser (Spectra-Physics, Santa Clara, CA) as a light source was introduced into the microscope (Zeiss Axiovert 200) by two tilting mirrors. The laser beam was focused on the back focal plane of the objective passing a 488-nm cleanup filter and a ZT488/647/780rpc-UF1 dichroic mirror (Chroma Technology GmbH, Olching, Germany). The sample signal was collected by the objective and filtered by a 535/70 emission filter (Chroma, Boston, MA). The data were acquired using a ProEM512 EMCCD camera (Princton Instruments, Trenton, NJ) with a 3-ms effective exposure time and 20 × 40 pixel acquisition area per measurement (pixel size 0.16 μm). ImFCS plugin for ImageJ software was used for data evaluation (http://www.dbs.nus.edu.sg/lab/BFL/imfcs_image_j_plugin.html). Detailed data analysis is described in Supplementary methods.

### Colony formation assay for the ATT experiment

CHO cells were submitted to HS twice. The first (priming) HS was conducted at different temperatures (40 °C, 42.5 °C, and 44 °C) for 20 min. After the priming HS, the cells were allowed to recover for 6 h at 37 °C, in line with other experiments. The second HS comprised a 20 min exposure to 46 °C. Immediately after the second HS, the cells were trypsinized, counted, and serially diluted with media before plating. The plated cells were incubated at 37 °C for 3–4 days. To visualize and assess colony formation, the cells were fixed in 4% paraformaldehyde, permeabilized with 2% Triton X-100, and stained with 500 nM propidium iodide (Molecular Probes). Imaging of the colonies was done by using Olympus SZX12 stereomicroscope (Olympus optical, Tokyo, Japan), with 530–560 nm excitation filter and 590–650 nm emission filter.

### Lipidomics

GPI-mGFP–expressing CHO cells were treated at specified temperatures for 20 min, washed twice with cold PBS, collected in Eppendorf tubes (1.4 × 10^6^ cells per tube), and centrifuged. The pellets were shaken in 1 mL of methanol containing 0.001% butylated hydroxytoluene as an antioxidant, for 10 min, and centrifuged at 10,000 × *g* for 5 min. The supernatant was transferred into a new Eppendorf tube and stored at −20 °C^13^.

Lipid standards were obtained from Avanti Polar Lipids (Alabaster, AL). The solvents used for extraction and for MS analyses were of liquid chromatographic grade (Merck, Darmstadt, Germany) and Optima LCMS grade from Thermo Scientific. All other chemicals were purchased from Sigma-Aldrich (Steinheim, Germany) and were of the best available grade.

MS analyses were performed using an LTQ-Orbitrap Elite instrument (Thermo Scientific, Bremen, Germany) equipped with a robotic nanoflow ion source TriVersa NanoMate (Advion BioSciences, Ithaca, NY). Lipid classes and species were annotated according to the lipid classification systems^72^, and the lipids were identified by LipidXplorer software^73^. Further measurement details are provided in the Supplementary methods. Lipidomics data are presented as the mean ± SD; statistical significance was determined by Student’s *t*-test and was accepted for p < 0.05. sPLS-DA and cluster analyses of lipidomics datasets were performed using MetaboAnalyst^74^.

### Data availability

All data generated or analysed during this study are included in this published article (and its Supplementary Information files) or available from the corresponding author on reasonable request.

## Electronic supplementary material

Supplementary Information
