# Supplementary material for: Mild heat induces a distinct “eustress” response in Chinese Hamster Ovary cells but does not induce heat shock protein synthesis
Source: Sci Rep. 2017 Nov 15;7:15643. doi: 10.1038/s41598-017-15821-8 (PMC5688065; doi:10.1038/s41598-017-15821-8)
Supplement: Supplementary file 1 — Supplementary Information [file 41598_2017_15821_MOESM1_ESM.pdf]

Supplementary Information

Mild heat induces a distinct "eustress" response in Chinese Hamster Ovary cells but does not induce heat shock protein synthesis

Begüm Peksel[1], Imre Gombos[1], Mária Péter[1], László Vigh, Jr.[1], Ádám Tiszlavicz[1], Mario Brameshuber[2], Gábor Balogh[1], Gerhard J. Schütz[2], Ibolya Horváth[1], László Vigh[1], Zsolt Török[1]*

[1]Institute of Biochemistry, Biological Research Centre of the Hungarian Academy of Sciences, Szeged H-6726, Hungary;
[2]Institute of Applied Physics – Biophysics, TU Wien, 1040 Vienna, Austria

*Corresponding author:
Zsolt Török,

Institute of Biochemistry, Biological Research Center, Hungarian Academy of Sciences, 6726-Szeged, Temesvari krt. 62, Hungary; Tel: +36-62-432038, E-mail: tzsolt@brc.hu

Supplementary Figures

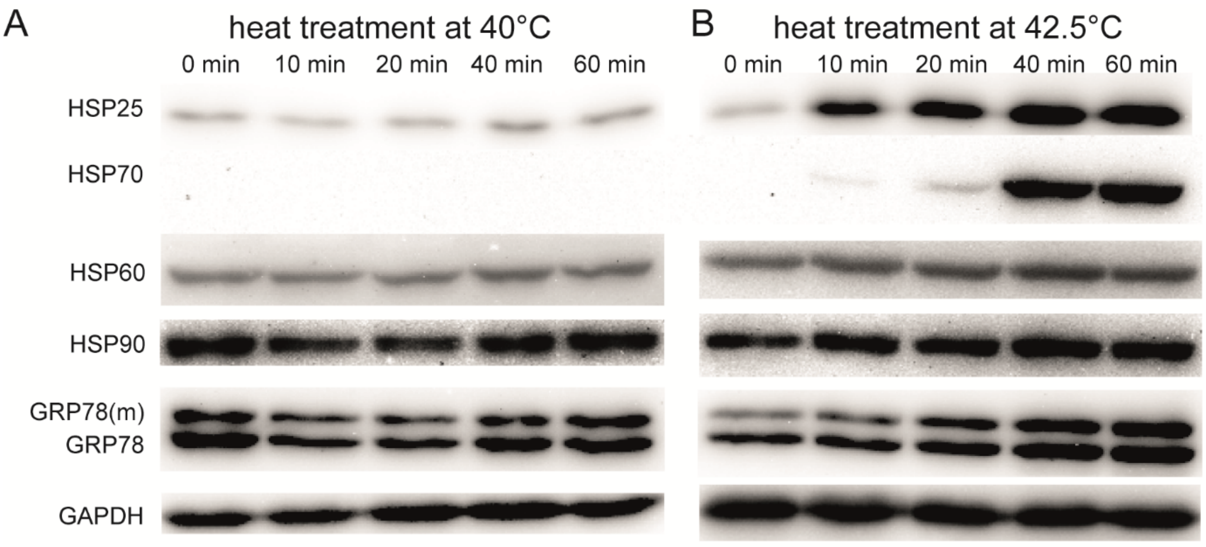

Supplementary Figure S1: The effect of HS duration on HSP induction. A GPI-mGFP–expressing CHO cell line was subjected to heat treatments at 40˚C (A) and 42.5˚C (B) for the specified time periods, and the samples were prepared for western blotting after 6 h of recovery at 37°C. Full lengths blots are presented in Supplementary Figure S7 and S8 for Figure S1A and S1B, respectively.

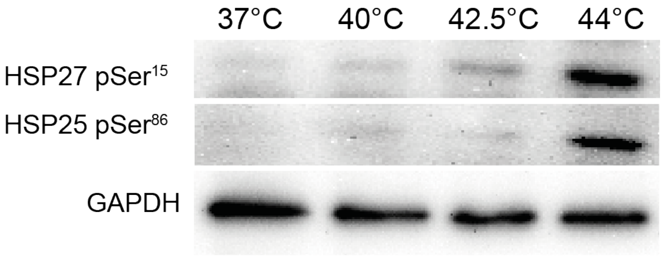

Supplementary Figure S2: The effect of heat treatment on the phosphorylation status of HSP25. CHO cells harboring the GPI-mGFP construct were incubated at a given temperature for 20 min before sample preparation for western blotting. See Supplementary Figure S9 for full length blots.

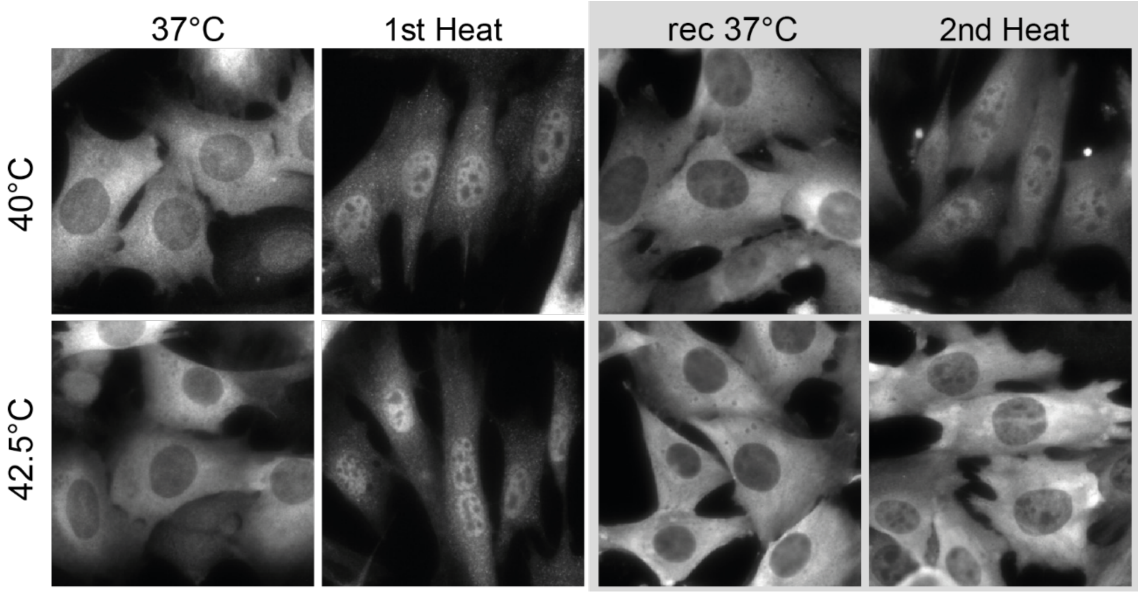

Supplementary Figure S3: HSP25 distribution after a second heat treatment of MEF cells. Representative images of HSP25 distribution in MEF cells at 37˚C; after a 20 min heat treatment at specified temperatures (1st heat); followed by 6 h recovery at 37˚C (rec 37˚C); and after a second, 20 min, heat treatment at specified temperatures (2nd heat).

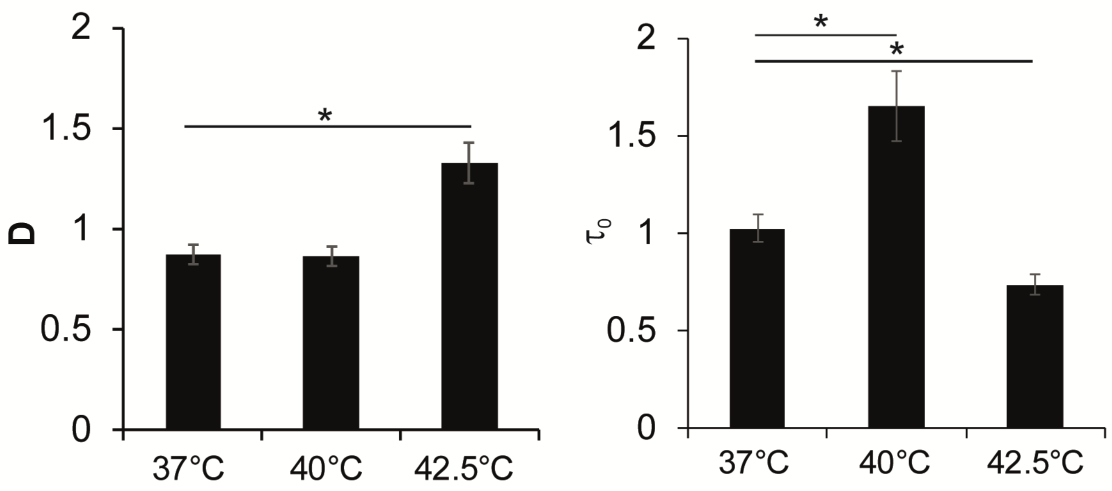

Supplementary Figure S4: The alteration of membrane diffusion of Bodipy FL-SM probe in live cells at different heat exposures. Changes in the diffusion coefficient (D) and confinement time (τ_0) of Bodipy FL-SM on the surface of wild-type CHO cells were measured by ImFCS. The values represent averages, and the error bars represent the standard error of mean (SEM) (n=9, $p<0.05$).

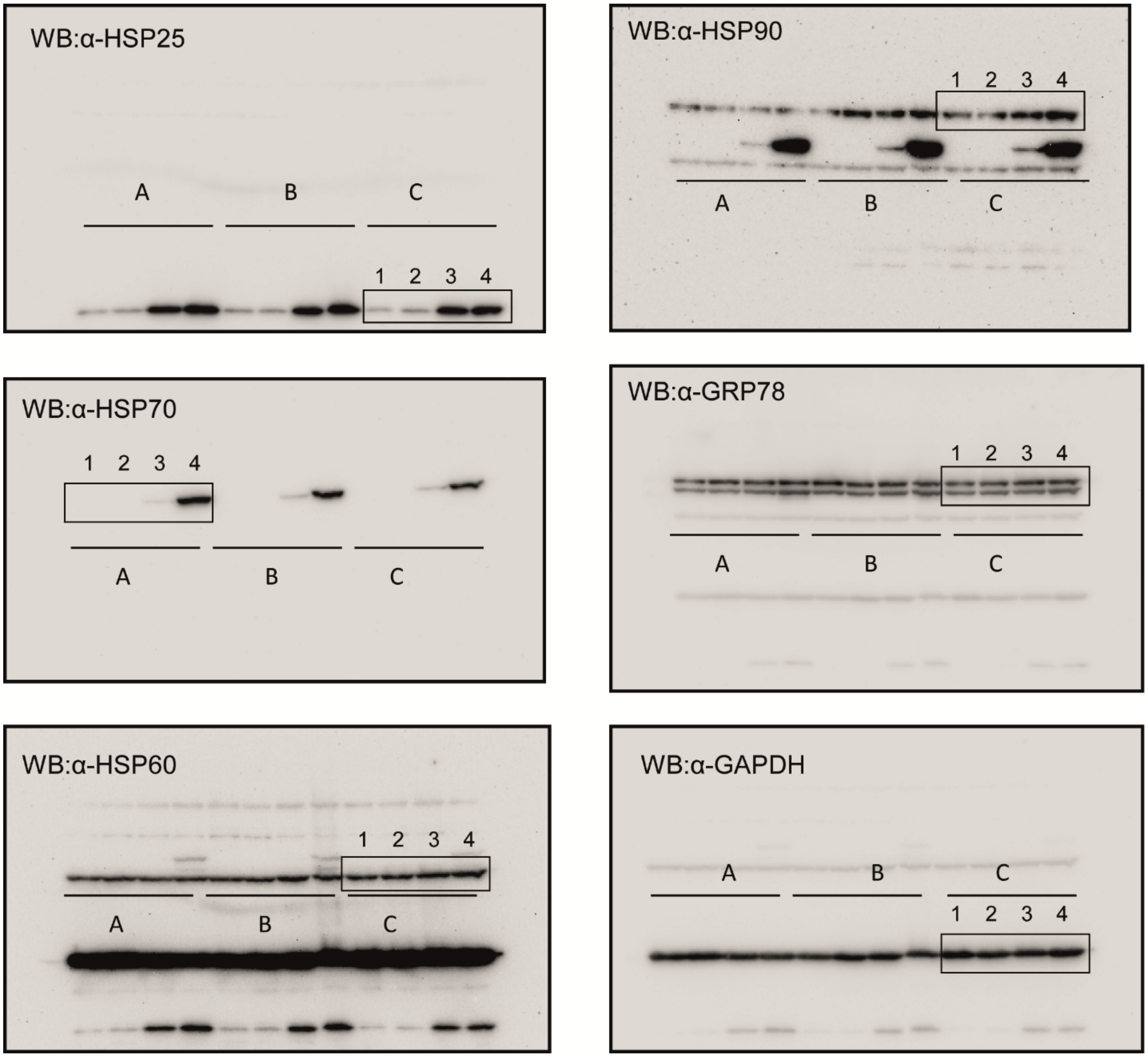

Supplementary Figure S5: Full Western blots of Figure 1A. The boxed image depicts what is shown in figure 1A. Blots were carried out on a single membrane with the following order of primary antibodies; HSP25, HSP70, HSP60 - GAPDH, HSP90 and GRP78. Treatments at 37°C (1), 40°C (2), 42.5°C (3) and 44°C (4) were shown in triplicates (A, B and C).

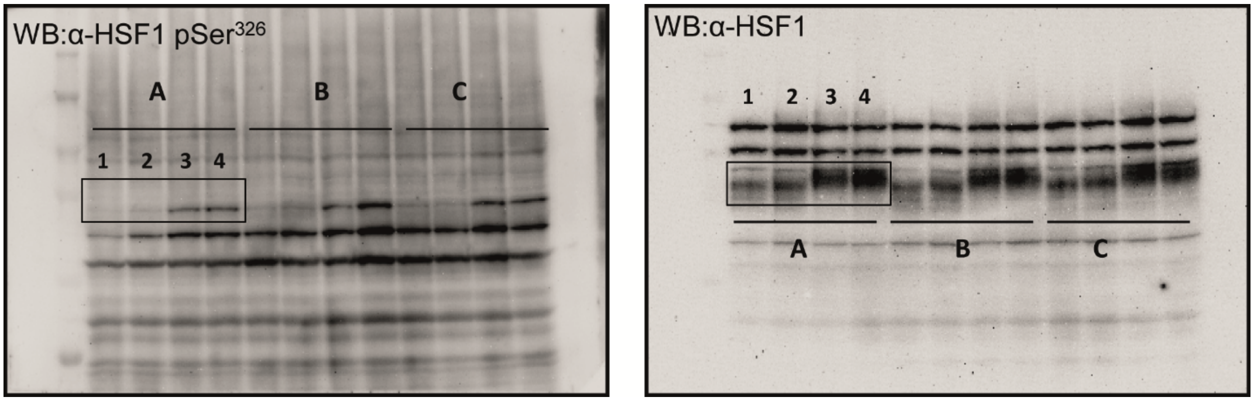

Supplementary Figure S6: Full Western blots of Figure 1B. The boxed image depicts what is shown in figure 1B. Treatments at 37°C (1), 40°C (2), 42.5°C (3) and 44°C (4) were shown in triplicates (A, B and C).

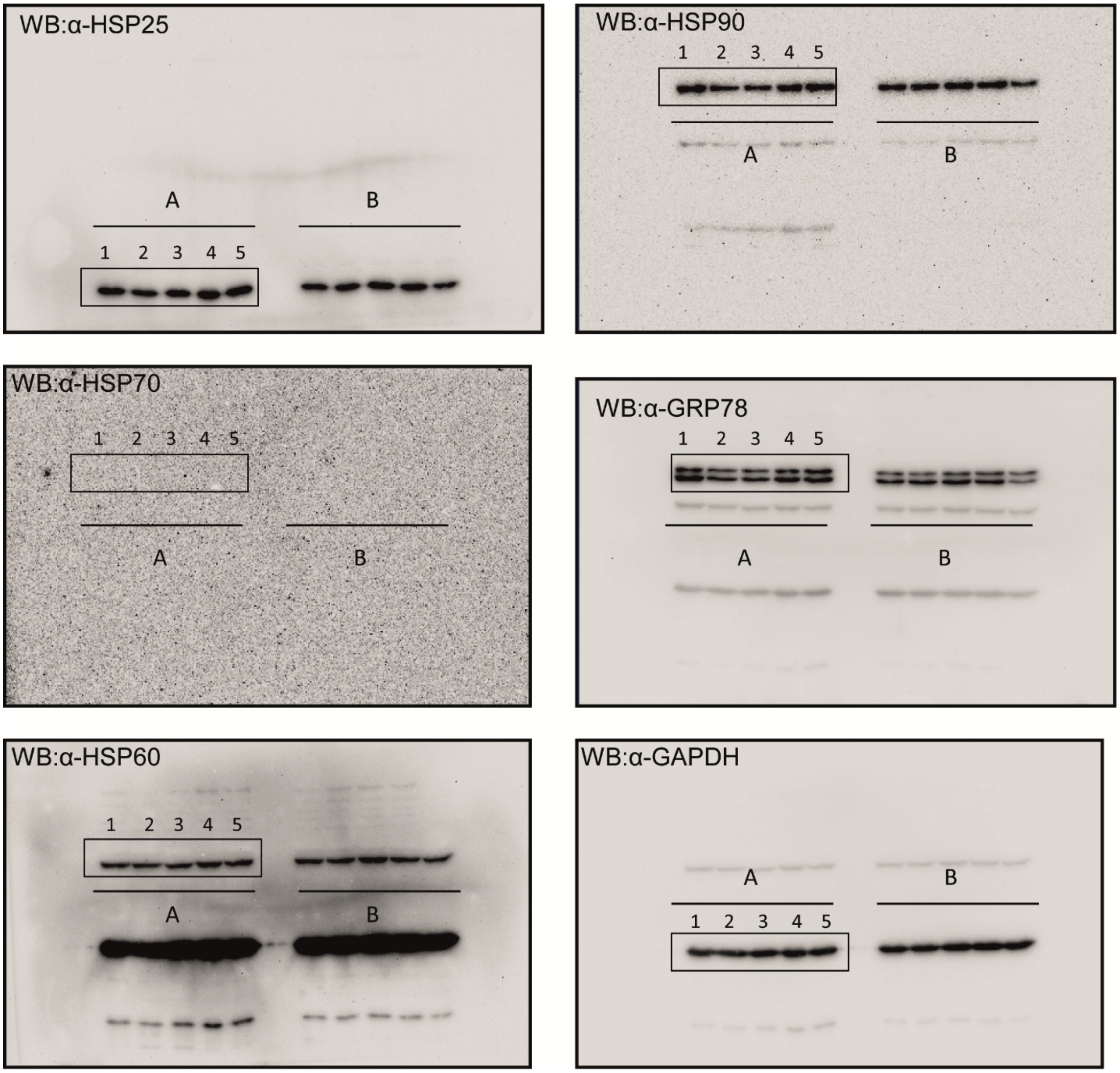

Supplementary Figure S7: Full Western blots of Supplementary Figure S1A. The boxed image depicts what is shown in Supplementary Figure S1A. Blots were carried out on a single membrane with the following order of primary antibodies; HSP25, HSP70, HSP60 - GAPDH, HSP90 and GRP78. Treatments at 0 min (1), 10 min (2), 20 min (3), 40 min (4) and 60 min (5) were shown in duplicates (A and B).

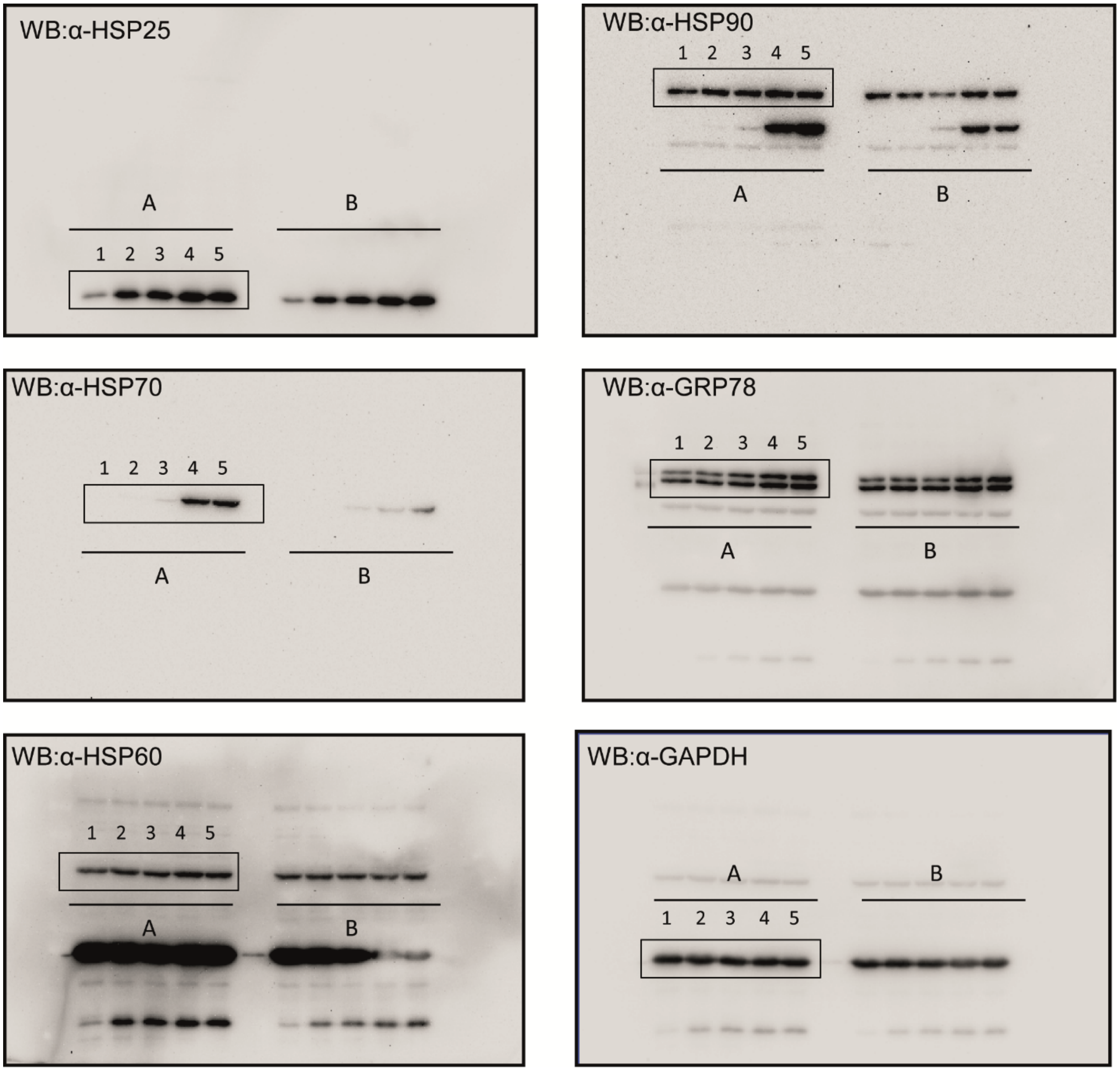

Supplementary Figure S8: Full Western blots of Supplementary Figure S1B. The boxed image depicts what is shown in Supplementary Figure S1B. Blots were carried out on a single membrane with the following order of primary antibodies; HSP25, HSP70, HSP60 - GAPDH, HSP90 and GRP78. Treatments at 0 min (1), 10 min (2), 20 min (3), 40 min (4) and 60 min (5) were shown in duplicates (A and B).

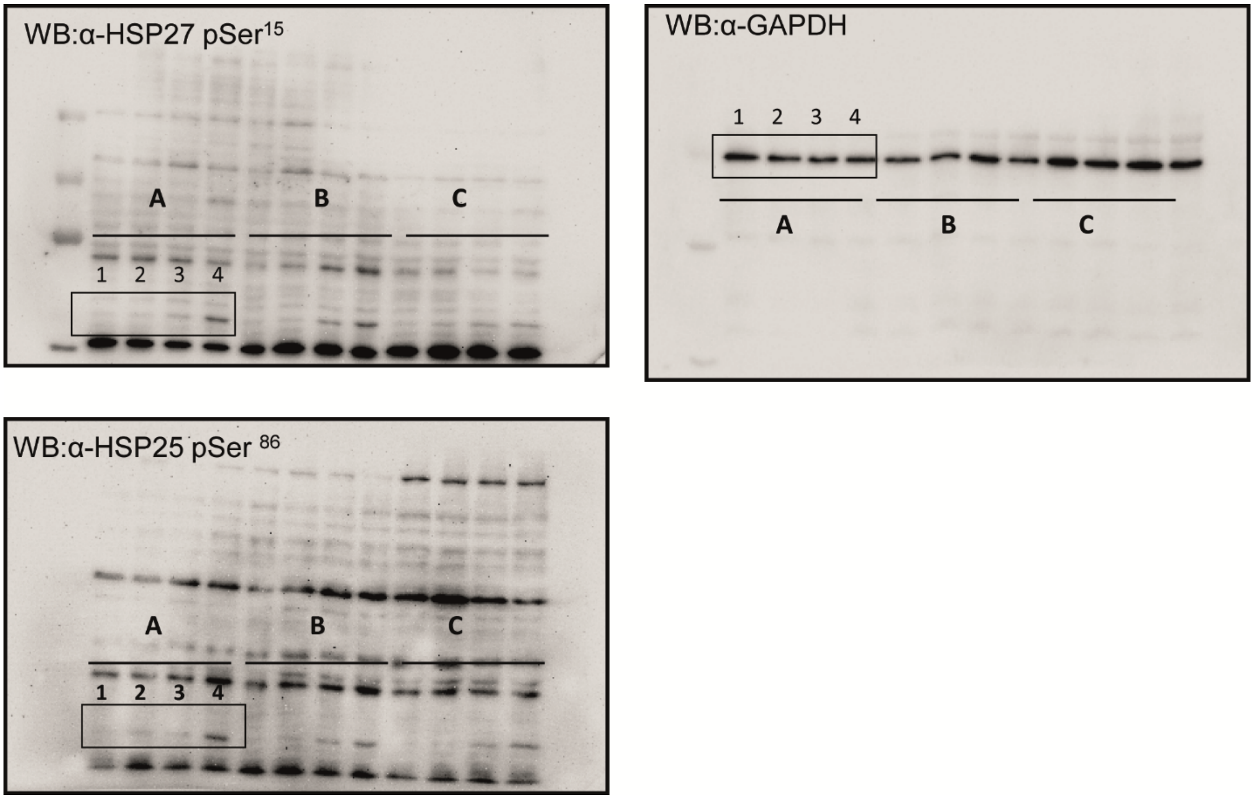

Supplementary Figure S9: Full Western blots of Supplementary Figure S2. The boxed image depicts what is shown in supplementary figure S2. Treatments at 37°C (1), 40°C (2), 42.5°C (3) and 44°C (4) were shown in triplicates (A, B and C).

Supplementary methods

ImFCS data analysis

The autocorrelation functions (ACFs) for every pixel were calculated using a multi-tau correlation scheme [91]. An exponential of polynomial bleach correction was used to correct data before fitting. To obtain the diffusion coefficient (D) for all pixels ACFs were fitted according to the equation below.

$$G(\tau) = \frac{1}{N} + \left[\frac{\operatorname{erf}\big(p(\tau)\big) + \frac{\left(e^{-(p(\tau))^2} - 1\right)}{\sqrt{\pi} p(\tau)}}{\operatorname{erf}\big(p(0)\big) + \frac{\left(e^{-(p(0))^2} - 1\right)}{\sqrt{\pi} p(0)}} \right]^2 + G_\infty p(\tau) = \frac{a}{\sqrt{4D\tau + \omega_0{}^2}}$$

Where $G(\tau)$ is the ACF, τ is the correlation time, N is the number of detected particles, *a* is the pixel size, and ω_0 is the $1/e^2$ radius of the Gaussian approximation of the point spread function. To identify and describe the mode of membrane organization by investigating the size-dependency of diffusion coefficient, we used the Imaging FCS type of FCS diffusion law [33]. According to that, the diffusion time (τ_D) of the fluorescent probe depends on the observation area (A_{eff}), as described by

$$\tau_D(A_{eff}) = \tau_0 + \frac{A_{eff}}{D}$$

where A_{eff} is the area of the membrane in which the labeled particle travels across, and is calculated by the convolution of the detection area with the point spread function. τ_0 is the intercept of the diffusion law plot on the y-axis of A_{eff}/D vs. A_{eff}. This parameter provides information about the diffusion confinement. The diffusion law can be plotted by using different A_{eff} values that are calculated by post-acquisition binning of pixels. In the case of free diffusion, D is constant regardless of A_{eff} so $\tau_D(A_{eff})$ is a straight line passing through the origin (±0.1 s) in the diffusion law graph. A heterogeneous system, where membrane domains or meshwork are present, however, allows spatial scale-dependent diffusion, which results in a remarkably different diffusion law plot with positive or negative intercepts for domain partitioning or meshwork diffusion, respectively [33].

Lipidomics methods

Lipidomics analyses were performed on an LTQ-Orbitrap Elite instrument (Thermo Fisher Scientific, Bremen, Germany) equipped with a robotic nanoflow ion source TriVersa NanoMate (Advion BioSciences, Ithaca, NY), using chips with 5.5-μm diameter spraying nozzles. The ion source was controlled by Chipsoft 8.3.1 software (Advion). The ionization voltages were +1.3 kV and −1.9 kV in the positive and negative modes, respectively, and the backpressure was set at 1 psi in both modes. The temperature of the ion transfer capillary was 330°C. Data acquisition was performed at the mass resolution $R_{m/z}$400=240,000.

Phosphatidylcholine (PC, diacyl, and PC-O, alkyl-acyl), lysophosphatidylcholine (LPC), and sphingomyelin (SM) were detected and quantified in the positive ion mode; phosphatidylethanolamine (PE, diacyl, and PE-Pl, alkenyl-acyl), lysophosphatidylethanolamine (LPE), phosphatidylinositol (PI), lysophosphatidylinositol (LPI), phosphatidylserine (PS), phosphatidic acid (PA), phosphatidylglycerol

(PG), cardiolipin (CL), ceramide (Cer), glucosyl ceramide (GlCer), and ganglioside (GM3) were detected and quantified in the negative ion mode.

For quantification, 10 μL of the lipid extract was diluted with 140 μL of the infusion solvent mixture (chloroform:methanol:iso-propanol, 1:2:1, by vol.) containing an internal standard mix (71 pmol PC d31-16:0/18:1, 25 pmol PE d31-16:0/18:1, 11 pmol PI d31-16:0/18:1, 19 pmol PS d31-16:0/18:1, 2.5 pmol PG d31-16:0/18:1, 1 pmol PA d31-16:0/18:1, 1.5 pmol CL 56:0, 5 pmol SM d18:1/17:0, 2 pmol Cer d18:1/17:0, 3 pmol GlCer d18:1/12:0 and 5 pmol GM3 d3-d18:1/18:0). Next, the mixture was split in two, and 5% (final concentration) dimethylformamide (additive for the negative ion mode) or 3 mM (final concentration) ammonium chloride (additive for the positive ion mode) were added to the sample halves. Each quantified lipid species accounted for more than 0.5% of the respective lipid class. The mass tolerance was 3 ppm. Data files generated by LipidXplorer queries were further processed by in-house Excel macros.

The sum of absolute mol% difference relative to control (SoamD score) values were calculated based on Tarasov et al, (2014), as follows:

$$\text{SoamD} = \Sigma \text{abs}(\text{mol\%}_{i,T} - \text{mol\%}_{i,37})$$

where $\text{mol\%}_{i,T}$ is the mol% of membrane lipids value for lipid species i at stress temperature T, and $\text{mol\%}_{i,37}$ is the mol% of membrane lipids value for lipid species i at control temperature (37°C).

Supplementary Table S1. Detailed lipidomic dataset

Data are expressed as mol% of membrane lipids and given as mean±SD, n=4;

* p<0.05, ** p<0.01 (vs. 37 °C)

Name	37 °C	40 °C	42.5 °C	44 °C
LPC [16:0]	0.356 ± 0.023	0.269 ± 0.039 **	0.168 ± 0.019 **	0.134 ± 0.020 **
LPC [18:1]	0.119 ± 0.034	0.074 ± 0.037	0.041 ± 0.022 **	0.030 ± 0.009 **
LPC [18:0]	0.132 ± 0.017	0.096 ± 0.014 *	0.064 ± 0.012 **	0.059 ± 0.008 **
**LPC**	0.606 ± 0.059	0.439 ± 0.062 **	0.273 ± 0.035 **	0.222 ± 0.030 **
PC [28:0]	0.177 ± 0.025	0.161 ± 0.015	0.147 ± 0.008	0.152 ± 0.015
PC [30:1]	0.325 ± 0.010	0.310 ± 0.006 *	0.315 ± 0.014	0.298 ± 0.019 *
PC [30:0]	1.853 ± 0.210	1.818 ± 0.097	1.781 ± 0.123	1.692 ± 0.129
PC [32:2]	0.360 ± 0.016	0.353 ± 0.018	0.350 ± 0.015	0.333 ± 0.014 *
PC [32:1]	4.664 ± 0.086	4.561 ± 0.075	4.392 ± 0.124 *	4.429 ± 0.065 **
PC [32:0]	3.372 ± 0.226	3.395 ± 0.294	3.554 ± 0.299	3.334 ± 0.212
PC [34:3]	0.338 ± 0.027	0.320 ± 0.014	0.321 ± 0.015	0.315 ± 0.023
PC [34:2]	4.097 ± 0.063	3.917 ± 0.060 **	3.877 ± 0.108 *	3.890 ± 0.109 *
PC [34:1]	19.814 ± 0.724	19.178 ± 0.309	18.993 ± 0.600	18.518 ± 0.407 *
PC [34:0]	0.380 ± 0.111	0.309 ± 0.043	0.330 ± 0.044	0.311 ± 0.038
PC [36:5]	0.401 ± 0.031	0.384 ± 0.016	0.371 ± 0.013	0.353 ± 0.016 *
PC [36:4]	2.160 ± 0.241	2.042 ± 0.068	1.993 ± 0.085	1.875 ± 0.092
PC [36:3]	1.688 ± 0.042	1.591 ± 0.034 *	1.574 ± 0.051 *	1.549 ± 0.057 **
PC [36:2]	7.242 ± 0.250	6.889 ± 0.185	6.824 ± 0.199 *	6.847 ± 0.180 *
PC [36:1]	1.826 ± 0.075	1.755 ± 0.049	1.730 ± 0.049	1.736 ± 0.041
PC [38:6]	1.168 ± 0.089	1.085 ± 0.031	1.089 ± 0.050	0.997 ± 0.054 *
PC [38:5]	1.640 ± 0.110	1.549 ± 0.039	1.540 ± 0.071	1.434 ± 0.078 *
PC [38:4]	1.022 ± 0.110	0.953 ± 0.056	0.958 ± 0.047	0.888 ± 0.065

PC [38:3]	0.425 ± 0.032	0.389 ± 0.011	0.400 ± 0.024	0.366 ± 0.016 *
PC [38:2]	0.486 ± 0.010	0.461 ± 0.010 *	0.466 ± 0.023	0.458 ± 0.009 **
PC [40:7]	0.439 ± 0.010	0.418 ± 0.008 *	0.416 ± 0.012 *	0.381 ± 0.008 **
PC [40:6]	0.514 ± 0.040	0.492 ± 0.023	0.499 ± 0.027	0.452 ± 0.032
PC [40:5]	0.349 ± 0.013	0.330 ± 0.008 *	0.341 ± 0.022	0.318 ± 0.029
**PC**	54.741 ± 2.105	52.659 ± 0.916	52.261 ± 1.764	50.925 ± 1.162 *
PC-O [30:0]	0.149 ± 0.009	0.156 ± 0.015	0.154 ± 0.020	0.167 ± 0.025
PC-O [32:1]	0.597 ± 0.021	0.576 ± 0.048	0.594 ± 0.060	0.612 ± 0.053
PC-O [32:0]	0.542 ± 0.046	0.501 ± 0.057	0.542 ± 0.096	0.567 ± 0.065
PC-O [34:2]	0.540 ± 0.065	0.564 ± 0.036	0.576 ± 0.058	0.585 ± 0.046
PC-O [34:1]	3.431 ± 0.115	3.326 ± 0.181	3.446 ± 0.177	3.591 ± 0.182
PC-O [34:0]	0.132 ± 0.013	0.137 ± 0.012	0.164 ± 0.024	0.170 ± 0.025 *
PC-O [36:6]	0.014 ± 0.011	0.026 ± 0.013	0.030 ± 0.003 *	0.032 ± 0.005 *
PC-O [36:5]	0.252 ± 0.092	0.299 ± 0.013	0.316 ± 0.029	0.303 ± 0.014
PC-O [36:4]	1.237 ± 0.069	1.158 ± 0.062	1.185 ± 0.131	1.164 ± 0.076
PC-O [36:3]	0.365 ± 0.056	0.371 ± 0.009	0.375 ± 0.025	0.389 ± 0.018
PC-O [36:2]	0.629 ± 0.033	0.610 ± 0.041	0.631 ± 0.048	0.641 ± 0.029
PC-O [36:1]	0.329 ± 0.034	0.331 ± 0.014	0.335 ± 0.020	0.359 ± 0.031
PC-O [38:6]	0.794 ± 0.059	0.812 ± 0.054	0.846 ± 0.090	0.834 ± 0.046
PC-O [38:5]	1.134 ± 0.060	1.120 ± 0.088	1.151 ± 0.138	1.134 ± 0.088
PC-O [38:4]	0.478 ± 0.050	0.439 ± 0.038	0.459 ± 0.066	0.449 ± 0.032
**PC-O**	10.620 ± 0.427	10.427 ± 0.621	10.802 ± 0.945	10.996 ± 0.564
LPE [18:1]	0.012 ± 0.003	0.010 ± 0.003	0.008 ± 0.001	0.006 ± 0.002 *
LPE [18:0]	0.020 ± 0.011	0.011 ± 0.003	0.007 ± 0.002	0.004 ± 0.002 *
LPE [22:6]	0.002 ± 0.002	0.008 ± 0.002 **	0.005 ± 0.002	0.005 ± 0.001 *
**LPE**	0.034 ± 0.015	0.029 ± 0.007	0.020 ± 0.002	0.015 ± 0.002 *
PE [32:1]	0.057 ± 0.012	0.060 ± 0.019	0.061 ± 0.009	0.074 ± 0.010

PE [34:2]	0.050 ± 0.016	0.102 ± 0.015 **	0.084 ± 0.028	0.101 ± 0.018 **
PE [34:1]	0.714 ± 0.059	0.768 ± 0.041	0.747 ± 0.069	0.812 ± 0.037 *
PE [36:4]	0.137 ± 0.007	0.194 ± 0.023 **	0.184 ± 0.030 *	0.179 ± 0.039
PE [36:3]	0.079 ± 0.004	0.092 ± 0.005 **	0.095 ± 0.019	0.097 ± 0.020
PE [36:2]	0.743 ± 0.074	0.797 ± 0.050	0.792 ± 0.098	0.853 ± 0.058
PE [36:1]	0.728 ± 0.083	0.807 ± 0.042	0.773 ± 0.081	0.841 ± 0.016 *
PE [38:6]	0.236 ± 0.026	0.252 ± 0.025	0.251 ± 0.034	0.273 ± 0.014 *
PE [38:5]	0.464 ± 0.042	0.489 ± 0.036	0.485 ± 0.059	0.506 ± 0.033
PE [38:4]	0.659 ± 0.027	0.705 ± 0.024 *	0.702 ± 0.058	0.730 ± 0.029 *
PE [38:3]	0.085 ± 0.011	0.092 ± 0.017	0.112 ± 0.023	0.118 ± 0.010 **
PE [38:2]	0.058 ± 0.007	0.072 ± 0.009 *	0.052 ± 0.026	0.074 ± 0.011
PE [40:7]	0.149 ± 0.005	0.204 ± 0.022 **	0.212 ± 0.025 **	0.231 ± 0.026 **
PE [40:6]	0.406 ± 0.059	0.473 ± 0.031	0.476 ± 0.049	0.510 ± 0.025 *
PE [40:5]	0.177 ± 0.011	0.211 ± 0.017 *	0.183 ± 0.014	0.220 ± 0.023 *
**PE**	4.740 ± 0.398	5.319 ± 0.280	5.210 ± 0.572	5.619 ± 0.239 **
PE-Pl [34:1]	0.316 ± 0.038	0.349 ± 0.048	0.343 ± 0.069	0.369 ± 0.033
PE-Pl [36:5]	0.084 ± 0.022	0.069 ± 0.007	0.073 ± 0.032	0.086 ± 0.025
PE-Pl [36:4]	0.467 ± 0.062	0.524 ± 0.085	0.512 ± 0.114	0.538 ± 0.046
PE-Pl [36:3]	0.031 ± 0.013	0.051 ± 0.005 *	0.048 ± 0.011	0.057 ± 0.004 **
PE-Pl [36:2]	0.142 ± 0.022	0.148 ± 0.021	0.152 ± 0.050	0.159 ± 0.015
PE-Pl [36:1]	0.150 ± 0.016	0.166 ± 0.040	0.176 ± 0.064	0.197 ± 0.028 *
PE-Pl [38:6]	0.599 ± 0.066	0.692 ± 0.083	0.697 ± 0.128	0.742 ± 0.057 *
PE-Pl [38:5]	0.328 ± 0.014	0.455 ± 0.063 **	0.426 ± 0.102	0.470 ± 0.036 **
PE-Pl [38:4]	0.339 ± 0.066	0.371 ± 0.079	0.354 ± 0.087	0.364 ± 0.027
PE-Pl [40:7]	0.099 ± 0.048	0.094 ± 0.028	0.093 ± 0.094	0.101 ± 0.055
PE-Pl [40:6]	0.173 ± 0.025	0.254 ± 0.049 *	0.227 ± 0.132	0.264 ± 0.055 *
PE-Pl [40:5]	0.147 ± 0.033	0.203 ± 0.037	0.208 ± 0.034 *	0.208 ± 0.017 *
PE-Pl [40:4]	0.087 ± 0.010	0.109 ± 0.013 *	0.111 ± 0.006 **	0.104 ± 0.022
**PE-Pl**	2.962 ± 0.248	3.485 ± 0.484	3.419 ± 0.828	3.658 ± 0.212 **

LPI [16:0]	0.017 ± 0.005	0.018 ± 0.007	0.020 ± 0.002	0.018 ± 0.003
LPI [18:1]	0.036 ± 0.009	0.040 ± 0.006	0.040 ± 0.003	0.040 ± 0.005
LPI [18:0]	0.078 ± 0.007	0.090 ± 0.014	0.086 ± 0.009	0.086 ± 0.015
**LPI**	0.131 ± 0.022	0.147 ± 0.021	0.146 ± 0.014	0.145 ± 0.023
PI [32:1]	0.049 ± 0.008	0.051 ± 0.005	0.054 ± 0.011	0.059 ± 0.009
PI [34:2]	0.095 ± 0.010	0.108 ± 0.012	0.113 ± 0.023	0.123 ± 0.009 **
PI [34:1]	0.654 ± 0.118	0.740 ± 0.092	0.723 ± 0.113	0.781 ± 0.061
PI [36:5]	0.056 ± 0.027	0.078 ± 0.023	0.077 ± 0.030	0.084 ± 0.024
PI [36:4]	0.417 ± 0.020	0.447 ± 0.032	0.448 ± 0.039	0.449 ± 0.026
PI [36:3]	0.116 ± 0.008	0.128 ± 0.015	0.127 ± 0.020	0.132 ± 0.012
PI [36:2]	0.582 ± 0.058	0.655 ± 0.033	0.624 ± 0.069	0.673 ± 0.066
PI [36:1]	0.264 ± 0.011	0.305 ± 0.013 **	0.311 ± 0.033 *	0.345 ± 0.035 **
PI [38:6]	0.079 ± 0.013	0.088 ± 0.014	0.088 ± 0.014	0.087 ± 0.011
PI [38:5]	0.365 ± 0.020	0.399 ± 0.032	0.394 ± 0.037	0.402 ± 0.036
PI [38:4]	4.928 ± 0.171	5.014 ± 0.374	5.057 ± 0.356	4.862 ± 0.289
PI [38:3]	0.269 ± 0.020	0.314 ± 0.035	0.305 ± 0.043	0.318 ± 0.037
PI [38:2]	0.017 ± 0.002	0.021 ± 0.001 *	0.017 ± 0.003	0.024 ± 0.007
PI [40:6]	0.167 ± 0.014	0.181 ± 0.018	0.180 ± 0.020	0.185 ± 0.010
PI [40:5]	0.211 ± 0.010	0.227 ± 0.019	0.230 ± 0.017	0.227 ± 0.012
PI [40:4]	0.118 ± 0.008	0.129 ± 0.015	0.125 ± 0.011	0.125 ± 0.006
**PI**	8.389 ± 0.344	8.886 ± 0.553	8.873 ± 0.805	8.877 ± 0.387
PS [32:1]	0.023 ± 0.004	0.023 ± 0.002	0.022 ± 0.002	0.023 ± 0.002
PS [34:2]	0.024 ± 0.003	0.019 ± 0.004	0.020 ± 0.003	0.020 ± 0.002
PS [34:1]	0.525 ± 0.042	0.552 ± 0.038	0.553 ± 0.072	0.574 ± 0.044
PS [36:3]	0.025 ± 0.003	0.023 ± 0.002	0.024 ± 0.003	0.025 ± 0.003
PS [36:2]	0.267 ± 0.020	0.302 ± 0.030	0.304 ± 0.037	0.326 ± 0.034 *
PS [36:1]	2.027 ± 0.134	2.169 ± 0.071	2.215 ± 0.152	2.269 ± 0.172

PS [38:6]	0.022 ± 0.003	0.018 ± 0.003	0.015 ± 0.002 **	0.013 ± 0.002 **
PS [38:5]	0.020 ± 0.003	0.031 ± 0.006 *	0.026 ± 0.004 *	0.030 ± 0.005 *
PS [38:3]	0.127 ± 0.018	0.161 ± 0.037	0.149 ± 0.022	0.176 ± 0.022 *
PS [38:2]	0.025 ± 0.004	0.034 ± 0.005 *	0.043 ± 0.006 **	0.038 ± 0.006 *
PS [38:1]	0.043 ± 0.003	0.047 ± 0.020	0.064 ± 0.009 **	0.054 ± 0.012
PS [38:0]	0.038 ± 0.019	0.066 ± 0.051	0.113 ± 0.036 *	0.072 ± 0.033
PS [40:6]	0.283 ± 0.020	0.324 ± 0.027	0.325 ± 0.025 *	0.344 ± 0.036 *
PS [40:5]	0.027 ± 0.027	0.098 ± 0.029 *	0.092 ± 0.029 *	0.096 ± 0.036 *
PS [40:4]	0.104 ± 0.018	0.125 ± 0.019	0.108 ± 0.028	0.118 ± 0.014
PS [40:2]	0.020 ± 0.005	0.023 ± 0.009	0.029 ± 0.011	0.031 ± 0.008
PS [40:1]	0.051 ± 0.004	0.068 ± 0.015	0.072 ± 0.010 **	0.079 ± 0.015 **
PS [42:2]	0.031 ± 0.002	0.039 ± 0.003 **	0.038 ± 0.001 **	0.041 ± 0.005 **
PS [42:1]	0.042 ± 0.008	0.051 ± 0.019	0.056 ± 0.014	0.066 ± 0.020
**PS**	3.724 ± 0.194	4.172 ± 0.177 *	4.267 ± 0.297 *	4.394 ± 0.229 **
PG [32:1]	0.001 ± 0.000	0.003 ± 0.001 **	0.003 ± 0.001 *	0.003 ± 0.001
PG [34:2]	0.010 ± 0.001	0.012 ± 0.004	0.014 ± 0.002 *	0.013 ± 0.003
PG [34:1]	0.308 ± 0.027	0.303 ± 0.025	0.296 ± 0.022	0.303 ± 0.013
PG [36:3]	0.032 ± 0.002	0.034 ± 0.004	0.033 ± 0.002	0.038 ± 0.004 *
PG [36:2]	0.190 ± 0.011	0.223 ± 0.008 **	0.218 ± 0.024	0.241 ± 0.020 **
PG [36:1]	0.153 ± 0.007	0.152 ± 0.012	0.162 ± 0.032	0.150 ± 0.011
PG [38:6]	0.005 ± 0.003	0.006 ± 0.002	0.007 ± 0.002	0.005 ± 0.002
PG [38:3]	0.006 ± 0.002	0.005 ± 0.001	0.007 ± 0.002	0.006 ± 0.002
PG [40:7]	0.093 ± 0.009	0.113 ± 0.007 *	0.113 ± 0.011 *	0.111 ± 0.007 *
PG [40:6]	0.011 ± 0.003	0.014 ± 0.004	0.014 ± 0.005	0.012 ± 0.003
PG [44:12]	0.008 ± 0.003	0.010 ± 0.004	0.010 ± 0.004	0.007 ± 0.002
**PG**	0.819 ± 0.028	0.875 ± 0.029 *	0.876 ± 0.024 *	0.889 ± 0.030 *
PA [32:1]	0.001 ± 0.001	0.001 ± 0.000	0.004 ± 0.001 **	0.002 ± 0.001
PA [34:2]	0.003 ± 0.002	0.004 ± 0.001	0.005 ± 0.001	0.005 ± 0.003

PA [34:1]	0.077 ± 0.004	0.068 ± 0.008	0.102 ± 0.005 **	0.115 ± 0.018 **
PA [36:2]	0.006 ± 0.004	0.010 ± 0.007	0.021 ± 0.006 **	0.029 ± 0.009 **
PA [36:1]	0.004 ± 0.001	0.005 ± 0.001	0.007 ± 0.002 *	0.007 ± 0.002
**PA**	0.091 ± 0.010	0.089 ± 0.013	0.138 ± 0.009 **	0.157 ± 0.023 **
CL [68:5]	0.013 ± 0.008	0.008 ± 0.000	0.020 ± 0.006	0.005 ± 0.001
CL [68:4]	0.037 ± 0.009	0.045 ± 0.011	0.033 ± 0.005	0.054 ± 0.009 *
CL [68:3]	0.026 ± 0.007	0.036 ± 0.010	0.035 ± 0.010	0.054 ± 0.007 **
CL [68:2]	0.042 ± 0.017	0.076 ± 0.030	0.073 ± 0.008 *	0.082 ± 0.029
CL [70:6]	0.053 ± 0.013	0.079 ± 0.009 *	0.079 ± 0.008 *	0.085 ± 0.005 **
CL [70:5]	0.114 ± 0.028	0.180 ± 0.010 **	0.172 ± 0.028 *	0.200 ± 0.006 **
CL [70:4]	0.091 ± 0.008	0.087 ± 0.016	0.093 ± 0.030	0.116 ± 0.008 **
CL [70:3]	0.027 ± 0.008	0.043 ± 0.007 *	0.045 ± 0.011 *	0.045 ± 0.005 **
CL [70:2]	0.015 ± 0.003	0.032 ± 0.015	0.036 ± 0.009 **	0.035 ± 0.009 **
CL [72:8]	0.008 ± 0.002	0.012 ± 0.004	0.011 ± 0.004	0.020 ± 0.001 **
CL [72:7]	0.026 ± 0.010	0.024 ± 0.008	0.024 ± 0.015	0.051 ± 0.002 **
CL [72:6]	0.095 ± 0.010	0.123 ± 0.007 **	0.123 ± 0.011 *	0.121 ± 0.042
CL [72:5]	0.047 ± 0.013	0.076 ± 0.005 **	0.068 ± 0.006 *	0.076 ± 0.003 **
CL [72:4]	0.028 ± 0.008	0.043 ± 0.006 *	0.049 ± 0.008 **	0.048 ± 0.002 **
CL [74:8]	0.017 ± 0.006	0.023 ± 0.014	0.016 ± 0.008	0.017 ± 0.009
CL [74:7]	0.042 ± 0.018	0.060 ± 0.006	0.064 ± 0.023	0.078 ± 0.005 **
CL [74:6]	0.028 ± 0.016	0.035 ± 0.009	0.042 ± 0.022	0.026 ± 0.007
**CL**	0.710 ± 0.129	0.982 ± 0.051 **	0.983 ± 0.111 *	1.112 ± 0.023 **
SM [34:2:2]	0.432 ± 0.067	0.454 ± 0.039	0.451 ± 0.022	0.471 ± 0.033
SM [34:1:2]	7.114 ± 0.484	7.083 ± 0.272	7.173 ± 0.230	7.390 ± 0.429
SM [40:1:2]	0.135 ± 0.022	0.145 ± 0.005	0.141 ± 0.017	0.154 ± 0.014
SM [42:2:2]	0.701 ± 0.128	0.710 ± 0.055	0.745 ± 0.043	0.769 ± 0.078
SM [42:1:2]	0.302 ± 0.022	0.277 ± 0.016	0.275 ± 0.005	0.277 ± 0.017
**SM**	8.685 ± 0.678	8.668 ± 0.369	8.786 ± 0.289	9.061 ± 0.551

Cer [34:1:2]	0.088 ± 0.002	0.112 ± 0.003 **	0.106 ± 0.006 **	0.113 ± 0.005 **
Cer [40:2:2]	0.001 ± 0.001	0.002 ± 0.000	0.002 ± 0.001	0.002 ± 0.000
Cer [40:1:2]	0.009 ± 0.002	0.013 ± 0.001 *	0.014 ± 0.002 *	0.017 ± 0.002 **
Cer [41:1:2]	0.003 ± 0.001	0.004 ± 0.002	0.004 ± 0.001	0.004 ± 0.001
Cer [42:2:2]	0.067 ± 0.002	0.086 ± 0.004 **	0.083 ± 0.007 **	0.094 ± 0.007 **
Cer [42:1:2]	0.057 ± 0.005	0.065 ± 0.006	0.067 ± 0.007	0.068 ± 0.008
**Cer**	0.226 ± 0.007	0.283 ± 0.014 **	0.276 ± 0.019 **	0.298 ± 0.018 **
GlCer [34:1:2]	0.144 ± 0.015	0.141 ± 0.002	0.155 ± 0.008	0.162 ± 0.007
GlCer [40:1:2]	0.009 ± 0.001	0.012 ± 0.002 *	0.009 ± 0.001	0.009 ± 0.003
GlCer [41:1:2]	0.001 ± 0.000	0.002 ± 0.001 *	0.003 ± 0.002	0.001 ± 0.000 *
GlCer [42:2:2]	0.032 ± 0.004	0.039 ± 0.002 *	0.039 ± 0.005 *	0.043 ± 0.005 *
GlCer [42:1:2]	0.026 ± 0.007	0.028 ± 0.003	0.030 ± 0.002	0.034 ± 0.004
**GlCer**	0.211 ± 0.019	0.222 ± 0.006	0.235 ± 0.009	0.249 ± 0.013 *
GM3 [34:1:2]	1.793 ± 0.085	1.749 ± 0.095	1.807 ± 0.091	1.771 ± 0.125
GM3 [40:1:2]	0.189 ± 0.019	0.172 ± 0.037	0.203 ± 0.021	0.182 ± 0.036
GM3 [41:2:2]	0.014 ± 0.003	0.010 ± 0.009	0.019 ± 0.006	0.015 ± 0.001
GM3 [42:2:2]	0.841 ± 0.041	0.900 ± 0.009 *	0.894 ± 0.012 *	0.908 ± 0.005 *
GM3 [42:1:2]	0.473 ± 0.018	0.489 ± 0.011	0.510 ± 0.021 *	0.507 ± 0.010 *
**GM3**	3.310 ± 0.152	3.319 ± 0.124	3.434 ± 0.093	3.383 ± 0.162
Class				
LPC	0.606 ± 0.059	0.439 ± 0.062 **	0.273 ± 0.035 **	0.222 ± 0.030 **
PC	54.741 ± 2.105	52.659 ± 0.916	52.261 ± 1.764	50.925 ± 1.162 *
PC-O	10.620 ± 0.427	10.427 ± 0.621	10.802 ± 0.945	10.996 ± 0.564
LPE	0.034 ± 0.015	0.029 ± 0.007	0.020 ± 0.002	0.015 ± 0.002 *
PE	4.740 ± 0.398	5.319 ± 0.280	5.210 ± 0.572	5.619 ± 0.239 **
PE-Pl	2.962 ± 0.248	3.485 ± 0.484	3.419 ± 0.828	3.658 ± 0.212 **

LPI	0.131 ± 0.022	0.147 ± 0.021	0.146 ± 0.014	0.145 ± 0.023
PI	8.389 ± 0.344	8.886 ± 0.553	8.873 ± 0.805	8.877 ± 0.387
PS	3.724 ± 0.194	4.172 ± 0.177 *	4.267 ± 0.297 *	4.394 ± 0.229 **
PG	0.819 ± 0.028	0.875 ± 0.029 *	0.876 ± 0.024 *	0.889 ± 0.030 *
PA	0.091 ± 0.010	0.089 ± 0.013	0.138 ± 0.009 **	0.157 ± 0.023 **
CL	0.710 ± 0.129	0.982 ± 0.051 **	0.983 ± 0.111 *	1.112 ± 0.023 **
SM	8.685 ± 0.678	8.668 ± 0.369	8.786 ± 0.289	9.061 ± 0.551
Cer	0.226 ± 0.007	0.283 ± 0.014 **	0.276 ± 0.019 **	0.298 ± 0.018 **
GlCer	0.211 ± 0.019	0.222 ± 0.006	0.235 ± 0.009	0.249 ± 0.013 *
GM3	3.310 ± 0.152	3.319 ± 0.124	3.434 ± 0.093	3.383 ± 0.162
